# Cardiometabolic Effects of a 12-Month, COVID-19 Lockdown-Interrupted Lifestyle Education Program for Arab Adolescents

**DOI:** 10.3389/fped.2022.887138

**Published:** 2022-06-09

**Authors:** Nasser M. Al-Daghri, Kaiser Wani, Malak N. K. Khattak, Abdullah M. Alnaami, Osama E. Amer, Naji J. Aljohani, Abdulaziz Hameidi, Hanan Alfawaz, Mohammed Alharbi, Shaun Sabico

**Affiliations:** ^1^Chair for Biomarkers of Chronic Diseases, Biochemistry Department, College of Science, King Saud University, Riyadh, Saudi Arabia; ^2^Obesity Endocrine and Metabolism Center, King Fahad Medical City, Riyadh, Saudi Arabia; ^3^Saudi Diabetes Charity, Riyadh, Saudi Arabia; ^4^Department of Food Science and Nutrition, College of Food Science and Agriculture, King Saud University, Riyadh, Saudi Arabia; ^5^Diabetes Centres and Units Administration, Ministry of Health, Riyadh, Saudi Arabia

**Keywords:** COVID-19 lockdown, metabolic syndrome, adolescents, lifestyle education, school

## Abstract

**Background:**

Childhood obesity and pediatric metabolic syndrome (MetS) have steadily increased during the last decade in Saudi Arabia. Intervention programs to prevent cardiometabolic disorders in Arab youth are needed.

**Objective:**

In this multi-school intervention study which was disrupted by COVID-19-imposed lockdowns (September 2019–April 2021), a 12-month lifestyle education program focused on improving the cardiometabolic status of Arab adolescents was investigated.

**Methods:**

A total of 2,677 Saudi students aged 12–18 years were recruited from 60 different secondary and preparatory year schools in Riyadh city, Saudi Arabia. The intervention was initially in-person counseling sessions and the subsequent sessions conducted virtually post-pandemic. Baseline anthropometrics and fasting blood samples for glucose, HbA1c, and lipid assessments were collected at baseline and after 12 months (704 participants).

**Results:**

Only 704 out of 2,677 (73.7% dropout) completed the intervention. At baseline, 19.6% of the participants were overweight and 18.1% were obese. A modest but significant decrease in the prevalence of central obesity [11.2 vs. 6.7% (−4.5% change, *p* = 0.002)], hypertension [22.3 vs. 11.4% (−10.9% change, *p* < 0.001)], and low-HDL cholesterol [61.6 vs. 23.3% (−38.3% change, *p* < 0.001)] was noted. Consequently, the prevalence of hypertriglyceridemia increased from 22.7 to 56.3% (+ 33.6%, *p* < 0.001) overtime. Also, the proportion of subjects who were able to change their status from MetS to non-MetS was significantly more in overweight/obese at baseline than normal weight (16.9 vs. 3.6%, adjusted OR = 3.42, *p* < 0.001).

**Conclusion:**

Interrupted lifestyle education programs secondary to COVID-19-imposed lockdowns still provided modest effects in improving cardiometabolic indices of Arab adolescents. Given the high digital literacy of Arab youth, improving the delivery of virtual lifestyle education programs may prove beneficial.

## Introduction

Overweight and obesity in children and adolescents have consistently increased in the last three decades and have emerged as a modern-day public health challenge (1). In the United States, data from the National Health and Nutrition Examination Survey indicated that obesity in youth aged 2–19 years increased from 13.9% in 1999–2000 to 18.5% in 2015–2016 (2). Unhealthy eating patterns and sedentary behavior have been mostly blamed for this increasing trend in childhood overweight and obesity (3, 4). The widespread pediatric obesity is also prevalent in Saudi Arabia and has been increasing to around 6.4% in 2002 and 9.3% in 2010, reaching 15.9% in 2015 (5). Following the discovery of oil in the 1960s, the Arabian Gulf States saw tremendous economic expansion, linked to a rise in the incidence of overweight and obesity (6). Obesity in adolescence and childhood is a risk factor for adult obesity, which is associated with chronic health complications such as type 2 diabetes, cardiovascular diseases, and metabolic syndrome (MetS) (7, 8).

MetS, a clinical low-grade inflammation state consisting of several cardiometabolic risk factors such as obesity, hypertension, dyslipidemia, and hyperglycemia, is common in obese adults but on a rising trend in adolescents in developing countries including Saudi Arabia (9, 10). Although cardiovascular events show up mostly in adults, the risk factors start developing earlier. Furthermore, the recent literature suggests that these risk factors, especially MetS, may be present silently in children and adolescents (11, 12). Therefore, effective intervention programs to reduce metabolic stress and life-threatening diseases should not be limited to high-risk groups such as obese adults. Still, they may be implemented as a prevention strategy in children and adolescents in a school setting. This is especially important in Saudi Arabia, where economic boom in the recent past has not only seen a parallel increase in the prevalence of obesity and metabolic syndrome but also because of the fact that the population of children and adolescents in Saudi Arabia is high with 31.3% of the total population under the age of 20 years, according to the semi-annual report of 2021 by the General Authority of Statistics, Kingdom of Saudi Arabia (13).

Earlier reviews of school-based lifestyle change interventions targeted at reducing the rising prevalence of obesity in adolescents yielded mixed results in terms of effectiveness (14, 15), and there have been very few studies on the effectiveness of lifestyle change programs in reducing different components of MetS in this age group, particularly in this part of the world with a surge in pediatric obesity in the past few decades. At the domestic level, education programs aimed at increasing awareness about vitamin D deficiency have been introduced, and while these programs modestly improve cardiometabolic profiles of adolescents, the primary outcome was the correction of micronutrient deficiency and not harder outcomes such as MetS and obesity (16, 17). This rising prevalence of obesity and associated metabolic disorders in adolescents are concerning. Hence, preventive measures based on the lifestyle education may prove beneficial in this population. In this context, the researchers at the Chair for Biomarkers in Chronic Diseases (CBCD), King Saud University (KSU), together with the Saudi Charitable Association of Diabetes (SCAD), initially designed an in-person, school-based 12-month intervention program where counseling about the benefits of good dietary habits and improved physical activity behavior was implemented. Unfortunately, this intervention program was interrupted following the nationwide COVID-19 lockdown in March 2020 but was eventually resumed, albeit virtually. In this study, thus, the efficacy of a 12-month lifestyle education program disrupted by the COVID-19 lockdown was investigated.

## Materials and Methods

### Study Participants and Baseline Assessment

A cluster-randomized school-based convenience sample educational interventional program was conducted in 60 high schools and preparatory year schools in Riyadh, Saudi Arabia. This was a 12-month lifestyle change educational program conducted by the Chair for Biomarkers of Chronic diseases (CBCD), King Saud University (KSU), in collaboration with the Saudi Charitable Association of Diabetes (SCAD) the program started in September 2019 and it lasted till March 2021. This program was designed to educate about the rising prevalence of obesity and MetS in children and adolescents in Saudi (10, 18). The study was approved by the institutional review board (IRB) of the College of Medicine, KSU, Saudi Arabia (no. E-19-4239, 29 October 2019), and the participants were recruited after obtaining parental informed consent. During the program, 2,677 school-attending children and adolescents from the age of 12–18 years initially agreed to participate. At recruitment, the participants were invited for a baseline assessment, which included an 8-h fasting blood sample withdrawal and anthropometrics to assess the status of BMI and different components of MetS before the inclusion of the study. This phase lasted for 5 months from the start of the program, and the entire baseline data were obtained before COVID-19-imposed lockdown in march 2020.

### Intervention

This 12-month counseling and educational health promotion initiative was centered on the importance of a balanced diet and physical exercise in a cohort of school-aged children and adolescents. The baseline assessment included orientation sessions where standardized health education into topics like healthy dietary habits such as reducing junk food and sugar-sweetened beverages and juices, and so on, encouraging reduction in portion sizes and discouraging sedentary behaviors. Besides, the participants were educated on the current rising prevalence of obesity and metabolic disorders and their adverse health effects in children. Also, the participants were educated about diabetes and its health effects, its prevention by reducing body weight by at least 5%, by learning about different constituents of daily caloric requirement and reducing fat intake and increasing the dietary fiber intake to at least 15 g/1,000 kcal. Emphasis was also laid on increasing physical activity, and participants were encouraged to at least devote 20 min/day of exercises of moderate intensity or activities such as swimming, cycling, walking, running, and so on. In addition, the program included the distributing educational materials (Supplementary Material 1) in pamphlets, booklets, infographics, videos, gamification, etc.

**FIGURE 1:**
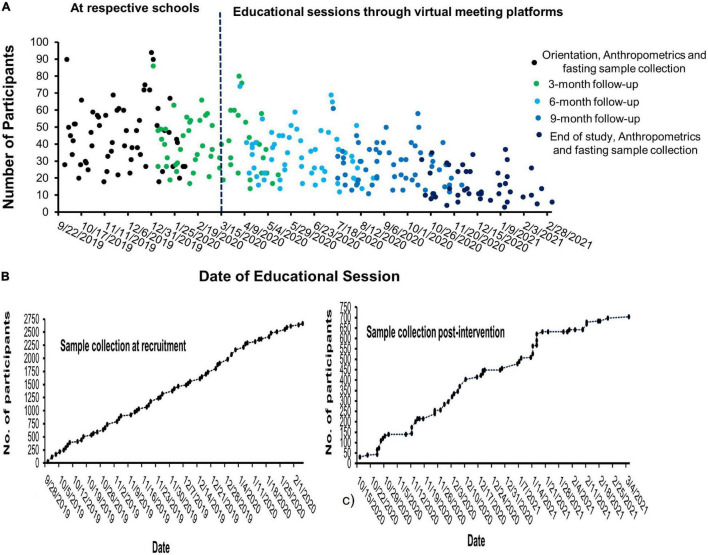
Time series graph depicting the recruitment of participants and the educational sessions provided during the study period. In panel **(A)**, the number of participants recruited per session at the respective schools and their follow-up through virtual meeting platforms during the emergency of COVID-19 is depicted while panels **(B)** and **(C)** depict the blood samples collected at recruitment and end of study, respectively.

The intervention program was designed to be imparted by health professionals in the form of a 20-min group educational session where lectures on eating healthy and being more physically active were delivered. Each participant was planned to be given five such educational sessions, one at baseline followed by 3 months of the intervening period. All the educational sessions were intended to be delivered in the respective schools; the baseline orientation was completed as planned; however, due to the emergence of COVID-19 and the resulting lockdown, most of the follow-up sessions were conducted through virtual meeting platforms such as Zoom, and social communications apps such as WhatsApp, Telegram, Facebook, Twitter, and so on. Therefore, the anthropometric data and the fasting blood samples of the study participants were collected at baseline and after 12 months post-intervention; baseline visit being completed in the respective schools whereas the 12-month data and samples were collected in selected health centers organized by the SCAD due to the emergence of COVID-19 infection and the closure of schools during the study period. The participants recruited during the study period are depicted in the form of a time series graph in Figure 1. Out of 2,677 participants recruited initially, only 704 agreed to visit for the delivery of fasting blood samples and post-intervention data at 12 months.

### Anthropometric Assessment, Sample Collection, and Biochemical Analysis

The anthropometric assessment and fasting blood sample collection were performed at baseline and 12 month post-intervention by trained research coordinators. The participants were informed before coming for delivering an 8-h fasting blood sample. The visit included an anthropometric assessment of weight (kg), height (cm), waist and hip circumferences (cm), and blood pressure (mm Hg) using standard methodology. Body mass index (BMI) was calculated as weight (kg) divided by height in meters. It was used to categorize the participants into normal, overweight, and obese using age and sex -specific cutoffs given by Cole et al. (19), which are equivalent to adult cutoffs of 25 and 30 kg/m2 for overweight and obesity, respectively.

The fasting blood samples collected at baseline and post-intervention were immediately transported to the CBCD laboratory, where they were aliquoted and stored at appropriate storing conditions for biochemical analysis. The biochemical analysis included assessing lipid profile and circulating glucose levels by standard routine kits by an automated biochemical analyzer, Konelab 20XT (Thermo Scientific, Vantaa, Finland). HbA1c was analyzed by the D-10 Hemoglobin Testing System (Bio-Rad Laboratories, California, United States). LIAISON XL, an automated quantitative analyzer, quantified serum 25 (OH) vitamin D levels (DiaSorin, Saluggia, Italy). It employs a sophisticated chemiluminescence approach with magnetic microparticle separation to achieve the highest sensitivity and accuracy of the test.

### Data Analysis

Anthropometric and biochemical data for baseline and post-intervention visits were used to check the status of full MetS and its risk components. The pediatric cutoff points for individual MetS components are different from adult cutoff points; however, the National Cholesterol Education Program’s (NCEP) MetS defining criteria of ≥3 MetS components (20) were used. The sex-specific pediatric cutoff points for individual MetS components were taken from the criteria given by Cook et al. (21), and these, as used in our data, are presented in Table 1.

**TABLE 1:** Pediatric definition of MetS.

MetS components	Cutoff	Boys						Girls					
		12	13	14	15	16	> 16	12	13	14	15	16	> 16
Central Obesity	WC (cm) ≥ 90th percentile	90	95.5	97	101.6	101.6	101.6	82	86	88	88.9	88.9	88.9
Hypertension	SBP (mmHg) ≥ 90th Percentile	126	130	130	130	130	130	130	130	130	130	130	130
	DBP (mmHg) ≥ 90th Percentile	85	85	85	85	85	85	85	85	85	85	85	85
Hypertriglyceridemia	TG ≥ 110 mg/dl or 1.24 mmol/L for 15 years below; TG ≥ 150 mg/dl or 1.7 mmol/L for 16 and above												
Low HDL-Cholesterol	HDL ≤ 40 mg/dl or 1.03 mmol/L												
Hyperglycemia	FBG ≥ 110 mg/dl or 6.1 mmol/L												
Full MetS	≥ 3 MetS components												

*The data at baseline were used to define the 90th percentile of waist circumference and blood pressure. Definition of Pediatric MetS based on Cook et al. (18). “WC” is waist circumference, “SBP” and “DBP” are systolic and diastolic blood pressure, respectively, “TG” is triglyceride, “HDL” is high-density lipoprotein, “FBG” is fasting blood sugar, and “MetS” is metabolic syndrome.*

Statistical Package for Social Sciences (SPSS) was used to examine the anthropometric and biochemical data and the status of MetS components in the participants at baseline and post-intervention (version 21, Armonk, NY, IBM). Frequencies (%) were used to represent the categorical variables. Continuous data were reported as mean and standard deviation (SD) for Gaussian variables and median (1st and 3rd quartiles) for non-Gaussian variables. Chi-square tests were used to compare the categorical variables. The independent Student’s *t*-test was used to compare groups at baseline, and the paired *t*-test was performed to compare mean differences between baseline and post-intervention visits. For non-Gaussian variables, the Mann–Whitney U test and the Wilcoxon test were utilized. The statistical significance was tested for two-tailed distributions at *p* < 0.05.

## Results

### Baseline Characteristics of Participants

At baseline, 2,677 Saudi adolescents were recruited for the study from 60 randomly chosen schools in Riyadh, Saudi Arabia (Table 2). These included 1,059 boys (39.6%) and 1,618 girls (60.4%). A large proportion (37.7%) of the total participants (44.2% boys and 33.5% girls) was overweight or obese. The adolescents recruited from primary and secondary schools were 61.6 (57.9% for boys and 64.1% for girls) and 38.4% (42.1% for boys and 35.9% for girls), respectively. The adolescents with MetS risk components were identified using the anthropometric and biochemical measurements according to the specialized criteria set by NCEP ATP III for them, and 256 (9.6% of total) met the criteria for full MetS. The prevalence of full MetS in boys and girls was 11.0 and 8.6%, respectively. Overall, among the individual components of MetS, low HDL-cholesterol was the most predominant (61.7%), and hyperglycemia (6.4%) was the least predominant. A similar trend in the predominance of the individual risk components of MetS was seen in both boys and girls as far as the baseline data were concerned.

**TABLE 2:** Characteristics of study participants at the time of recruitment.

	All (2,677)	Boys (1,059)	Girls (1,618)
**Educational status**			
Primary	1,650 (61.6)	613 (57.9)	1,037 (64.1)
Secondary	1,027 (38.4)	446 (42.1)	581 (35.9)
BMI Status*			
Normal	1,667 (62.3)	591 (55.8)	1,076 (66.5)
Overweight	526 (19.6)	204 (19.3)	322 (19.9)
Obese	484 (18.1)	264 (24.9)	220 (13.6)
**Anthropometrics**			
Age (years)	14.76 ± 1.7	14.89 ± 1.6	14.68 ± 1.7
Weight (Kg)	57.25 ± 18.6	61.89 ± 21.9	54.22 ± 15.4
Height (cm)	156.38 ± 10.7	159.45 ± 11.3	154.37 ± 9.8
BMI (Kgm^–2^)	23.34 ± 7.2	24.1 ± 7.6	22.85 ± 6.9
Waist (cm)	73.59 ± 17	74.62 ± 23	72.92 ± 11.6
Hips (cm)	89.00 ± 18.9	84.38 ± 25	92.01 ± 12.5
Systolic BP (mm HG)	118.6 ± 16.9	119.45 ± 14.8	118.04 ± 18.1
Diastolic BP (mm HG)	72.35 ± 12.5	67.8 ± 10.2	75.33 ± 13
**Biochemical characteristics**			
Cholesterol (mmol/l)	4.4 ± 0.8	4.33 ± 0.8	4.45 ± 0.8
HDL-Chol (mmol/l)	0.99 ± 0.2	1.00 ± 0.2	0.98 ± 0.2
Triglycerides (mmol/l)	0.96 (0.8,1.3)	1.03 (0.8,1.4)	0.93 (0.8,1.2)
FBG (mmol/l)	5.23 ± 1.2	5.31 ± 1.1	5.17 ± 1.2
HbA1c	5.10 ± 0.7	5.25 ± 0.7	5.00 ± 0.7
25 (OH) D (nmol/l)	29.8 (22.2,39.4)	36.1 (29,45.8)	25.6 (20,34)
**MetS components**			
Central Obesity	286 (10.7)	111 (10.5)	175 (10.8)
Hypertension	602 (22.5)	213 (20.1)	389 (24.0)
Hypertriglyceridemia	625 (23.3)	298 (28.1)	327 (20.2)
Low HDL_Cholesterol	1,652 (61.7)	598 (56.5)	1,054 (65.1)
Hyperglycemia	170 (6.4)	81 (7.6)	89 (5.5)
Full MetS	256 (9.6)	117 (11.0)	139 (8.6)
MetS Components (N)	1.25 ± 0.9	1.23 ± 1.1	1.26 ± 0.9

Data for categorical variables are presented as frequency (%) while for continuous variables are presented either as mean ± standard deviation or as median (quartile 1, quartile 3); *Based on the definition of Cole et al. (17), “BMI” is body mass index and “FBG” is fasting blood sugar.

### Changes in Clinical Characteristics Post-intervention

From the total of 2,677 adolescents who provided questionnaire data and blood samples at the beginning of the study, 1,973 (73.7%) of the subjects either lost to follow-up, discontinued, or did not consent for a follow-up fasting blood sample appointment at various stages due to COVID-19 situation prevalent during the study program. The final analysis was done for the remaining 704 adolescents (47.0% boys and 55.8% girls) for whom all of the data necessary to evaluate MetS pre- and post-intervention were collected (Table 3). Overall, there was an insignificant decrease of 3.3% in overweight or obesity percentage from baseline to end of the study. A modest but statistically significant improvement in BMI (*p* = 0.017) was seen post-intervention in all subjects; however, the significance disappeared when data were seen individually for either sex. Statistically significant increase in lipids (*p* < 0.001 for total cholesterol, HDL-cholesterol, and triglycerides) was accompanied by an increase in fasting glucose levels (*p* < 0.001) post-intervention when all subjects. A similar trend could be seen in either sex. Circulating vitamin D levels also increased post-intervention when data from all subjects were analyzed; however, when data were looked at individually for sexes, this significant increase in vitamin D levels was seen only in females (*p* < 0.001).

**TABLE 3:** Change in anthropometry and biochemical characteristics for those who completed the lifestyle change educational program.

	All (704)			Boys (331)			Girls (373)		
				Baseline	Follow-up		Baseline	Follow-up	
**BMI status**									
Overweight or Obese	261 (37.1)	238 (33.8)	0.07	151 (45.6)	141 (42.6)	0.453	110 (29.5)	97 (26.0)	0.287
**Anthropometrics**									
Age (years)	14.91 ± 1.7			14.81 ± 1.6			14.99 ± 1.8		
Weight (kg)	57.97 ± 20.1	59.85 ± 17.9	< 0.001	62.87 ± 23.8	64.86 ± 21.2	< 0.001	53.62 ± 14.8	55.4 ± 12.8	< 0.001
Height (cm)	156.62 ± 1.3	160.52 ± 11.3	< 0.001	159.45 ± 11.3	162.56 ± 11.9	< 0.001	154.71 ± 10.2	158.72 ± 10.9	< 0.001
BMI (kg/m^2^)	23.54 ± 7.8	23.25 ± 6.7	0.017	24.61 ± 8.2	24.38 ± 7.1	0.131	22.59 ± 7.3	22.25 ± 6.3	0.066
Waist (cm)	75.54 ± 16.2	77 ± 14.9	0.006	79.82 ± 19.7	86.2 ± 13.3	< 0.001	71.75 ± 11	68.83 ± 11.1	< 0.001
Hips (cm)	90.14 ± 17.4	85.25 ± 15.7	< 0.001	89.14 ± 21.5	91.47 ± 14.4	0.009	91.03 ± 12.8	79.74 ± 14.8	< 0.001
Systolic BP (mm HG)	119.78 ± 16.3	111.18 ± 13.6	< 0.001	119.47 ± 14.8	117.63 ± 9	0.034	120.06 ± 17.5	105.45 ± 14.3	< 0.001
Diastolic BP (mm HG)	71.98 ± 11.9	72.71 ± 8.4	0.161	67.76 ± 9.5	73.17 ± 9.7	< 0.001	75.72 ± 12.5	72.3 ± 7	< 0.001
**Biochemical characteristics**									
Cholesterol (mmol/l)	4.44 ± 0.8	6.23 ± 1.6	< 0.001	4.4 ± 0.8	6.01 ± 1.4	< 0.001	4.48 ± 0.8	6.42 ± 1.7	< 0.001
HDL-Chol (mmol/l)	0.99 ± 0.2	1.57 ± 0.6	< 0.001	1.01 ± 0.2	1.35 ± 0.5	< 0.001	0.97 ± 0.2	1.76 ± 0.7	< 0.001
Triglycerides (mmol/l)	0.95 (0.8,1.3)	1.53 (1.1,2.1)	< 0.001	1.03 (0.8,1.3)	1.63 (1.1,2.2)	< 0.001	0.91 (0.7,1.1)	1.46 (1,1.9)	< 0.001
FBG (mmol/l)	5.24 ± 0.9	5.66 ± 2.8	< 0.001	5.33 ± 1.2	5.79 ± 2.6	0.005	5.16 ± 0.7	5.54 ± 2.9	0.01
HbA1c	5.11 ± 0.6	5.32 ± 1.3	< 0.001	5.23 ± 0.6	5.24 ± 1.2	0.679	5.00 ± 0.6	5.38 ± 1.3	< 0.001
25 (OH) D (nmol/l)	30.7 (22.9,39.5)	38.7 (25.1,58.5)	< 0.001	35.6 (28.8,46.2)	34.6 (24.4,49.5)	0.253	25.7 (19.8,34)	41.55 (25.6,63.1)	< 0.001

*Data for categorical variables are presented as frequency (%) while for continuous scalar variables are presented either as mean ± standard deviation or as median (quartile 1, quartile 3). Relevant statistical tests were employed to check the differences pre- and post-intervention. p < 0.05 was considered statistically significant. “BMI” is body mass index and “FBG” is fasting blood sugar.*

### Change in MetS and Its Individual Components Post-intervention

There was an insignificant decrease of 1.7% from 9.5% at baseline to 7.8% intervention for the prevalence of full MetS when data were analyzed for all subjects, and it did not change significantly for either sex (Table 4). However, after an investigation into individual components that sum up to full MetS, a modest but statistically significant decrease in the prevalence was noted in central obesity (4.5%, *p* = 0.002) and hypertension (10.9%, *p* < 0.001) and a significant parallel increase in hyperglycemia (12.2%, *p* < 0.001). The components that showed maximum changes in the prevalence post-intervention were low-HDL cholesterol which decreased from 61.6 to 23.3% (a decrease of 38.3%, *p* < 0.001) and hypertriglyceridemia, which increased from 22.7 to 56.3% post-intervention (an increase of 33.6%, *p* < 0.001). Similar trends were seen when data were analyzed for different sexes.

**TABLE 4:** Prevalence of MetS and its individual components in Saudi adolescent’s pre- and post-intervention.

	Overweight or Obese	Central Obesity	Hypertension	Hyper triglyceridemia	Low HDL-Cholesterol	Hyperglycemia	Full MetS	MetS Components (*N*)
**All subjects (704)**								
Baseline	261 (37.1)	79 (11.2)	157 (22.3)	167 (22.7)	434 (61.6)	53 (7.5)	67 (9.5)	1.26 ± 0.9
Follow-up	238 (33.8)	47 (6.7)	80 (11.4)	396 (56.3)	164 (23.3)	139 (19.7)	55 (7.8)	1.12 ± 0.9
Change	3.3 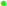	4.5 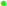	10.9 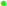	33.6 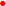	38.3 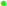	12.2 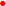	1.7 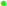	0.14 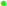
*p*	0.07	0.002	< 0.001	<0.001	< 0.001	<0.001	0.238	0.002
**Boys (331)**								
Baseline	151 (45.6)	48 (14.5)	64 (19.3)	95 (28.7)	179 (54.1)	28 (8.5)	39 (11.8)	1.25 ± 1.1
Follow-up	141 (42.6)	28 (8.5)	61 (18.4)	199 (60.1)	106 (32)	69 (20.8)	45 (13.6)	1.32 ± 0.9
Change	3.0 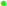	6.0 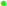	0.9 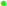	31.4 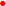	22.1 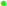	12.3 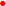	1.8 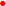	0.07 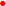
*p*	0.453	0.015	0.769	< 0.001	<0.001	< 0.001	0.487	0.337
**Girls (373)**								
Baseline	110 (29.5)	31 (8.3)	93 (24.9)	72 (19.3)	255 (68.4)	25 (6.7)	28 (7.5)	1.28 ± 0.9
Follow-up	97 (26.0)	19 (5.1)	19 (5.1)	197 (52.8)	58 (15.5)	70 (18.8)	21 (5.6)	0.93 ± 0.8
Change	3.5 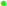	3.2 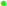	19.8 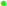	33.5 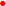	52.9 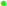	12.1 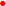	1.9 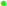	0.35 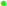
*p*	0.287	0.08	< 0.001	<0.001	< 0.001	<0.001	0.301	< 0.001

*Data are presented as frequency (%). Relevant statistical tests were employed to check the differences in the prevalence pre- and post-intervention. 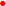 Depicts an increase while and 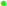 depicts a decrease in prevalence post-intervention. p < 0.05 was considered statistically significant.*

### Prevalence of Positive Change in the Status of MetS and Its Components Post-intervention

**FIGURE 2:**
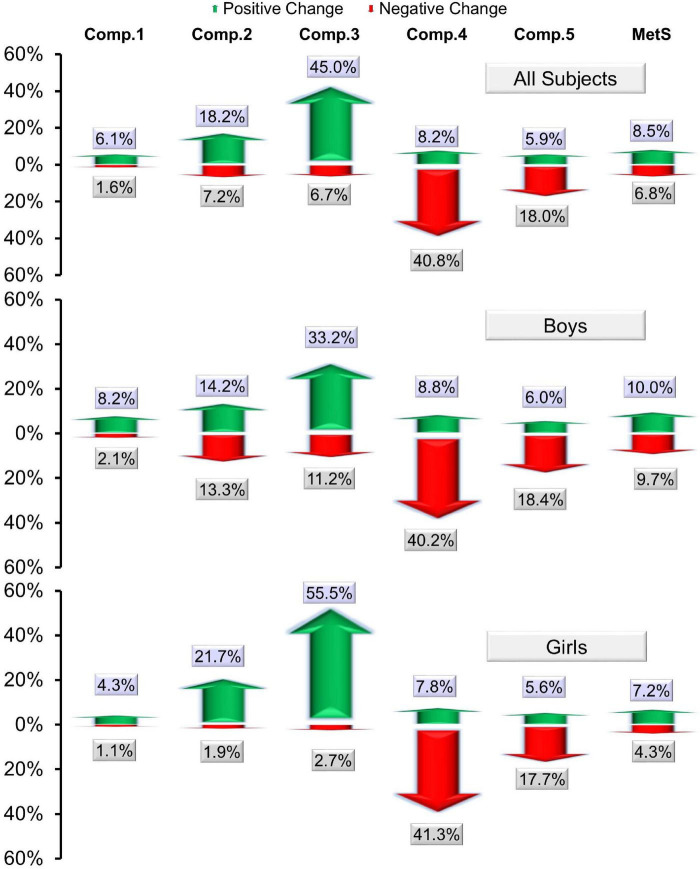
A bar graph showing the percentages of positive change (from “yes” at baseline to “no” post-intervention) and negative change (from “no” at baseline to “yes” post-intervention) in MetS and its components. Here, comp. 1, 2, 3, 4, and 5 represent “central obesity,” “hypertension,” “low-HDL cholesterol,” “hypertriglyceridemia,” and “hyperglycemia,” respectively.

The prevalence of subjects in which the status of MetS and its components changed from “yes” at baseline to “no” post-intervention was tabulated as Table 5. The percentages of positive change (from “yes” at baseline to “no” post-intervention) and negative change (from “no” at baseline to “yes” post-intervention) were plotted as bar graphs for all subjects and both sexes in Figure 2.

**TABLE 5:** Age-adjusted odds of the positive change in status of MetS and its components post intervention in participants with “overweight/obese” compared to the normal.

MetS_components	Baseline	Follow-up	All (704)	Overweight/obese (B) (261)	Normal weight (B) (443)	Normal weight (B)	Overweight/obese (B)			
							O.R. (95% C.I.)	*p*	O.R. (95% C.I.)^a^	*p* ^a^
Central Obesity	Yes	No	43 (6.1)	43 (16.5)	0 (0.0)	Ref.	-	-	-	-
Hypertension	Yes	No	128 (18.2)	68 (26.1)	60 (13.5)	Ref.	2.25 (1.5–3.3)	< 0.01	2.28 (1.5–3.4)	< 0.01
Hypertriglyceridemia	Yes	No	58 (8.2)	28 (10.7)	30 (6.8)	Ref.	1.65 (1.0–2.8)	0.07	1.70 (1.0–2.9)	0.06
Low HDL-Cholesterol	Yes	No	317 (45)	131 (50.2)	186 (42.0)	Ref.	1.39 (1.0–1.9)	0.035	1.36 (1.0–1.9)	0.04
Hyperglycemia	Yes	No	41 (5.8)	19 (7.3)	22 (5.0)	Ref.	1.50 (0.8–2.8)	0.21	1.48 (0.8–2.8)	0.23
Subjects where status of at least one MetS components improved			426 (60.5)	187 (71.6)	239 (53.9)	Ref.	2.16 (1.6–3.0)	< 0.01	2.14 (1.5–3.0)	< 0.01
Full MetS	Yes	No	60 (8.5)	44 (16.9)	16 (3.6)	Ref.	5.41 (2.9–9.8)	< 0.01	3.42 (1.8–6.5)^b^	< 0.01^b^

*Data presented as frequency (%) represents the proportions of individuals where status changed from “yes” to “no” post intervention. Bivariate regression analysis was carried out to check the odds of these proportions in “overweight/obese at baseline” compared to “normal weight at baseline.” Superscripts “a” and “b” represents, respectively, “adjusted with age” and “adjusted with age and central obesity component.” p < 0.05 was considered statistically significant.*

Bivariate regression analysis in this status between the baseline status of overweight/obese and normal weight revealed a statistically significant improvement in those who were overweight/obese at baseline compared to those who had normal weight (16.9 vs. 3.6%, adjusted OR of full MetS = 3.42, 95% CI 1.8–6.5, *p* < 0.01). Additionally, the positive change in status of MetS components hypertension (26.1 vs. 13.5%, adjusted OR = 2.28 and *p* < 0.01) and low HDL-cholesterol (50.2 vs. 42.0%, adjusted OR = 1.36 and *p* = 0.04) was significantly higher in participants who were overweight/obese at baseline compared to normal weight. Furthermore, when the proportion of subjects was compared between these groups where the status of at least one MetS component changed from “yes” to “no” post-intervention, a statistically significant improvement was seen in those who were overweight/obese at baseline compared to normal weight (71.6 vs. 53.9%, adjusted O.R. = 2.14, *p* < 0.01).

## Discussion

This school-based lifestyle intervention and counseling program, aimed at mitigating the growing prevalence of metabolic disorders in children and adolescents in Saudi Arabia, suggested its effectiveness in improving the cardiometabolic indices, particularly the components of MetS such as low-HDL cholesterol, hypertension, and central obesity. The status of full MetS showed an insignificant overall decrease of 1.7% from baseline; however, 60 (8.5%) of the total 704 participants who completed the program were able to change the status of MetS at baseline to non-MetS post-intervention. This is taking into full consideration that the COVID-19 lockdown disrupted the intervention program. The effectiveness of the intervention may also be determined by the fact that in 426/704 (60.5%) of the individuals, at least one of the five MetS risk components changed from being present at baseline to being absent post-intervention. This is heartening because this sort of multi-platform counseling intervention is feasible to be delivered in school settings and may be productive, especially in a population such as Saudi Arabia where economic upsurge from last five decades has had a parallel surge in cardiometabolic risk factors such as obesity and MetS in children and adolescents.

The prevalence of 37.7% overweight/obesity in Saudi adolescents from total of 2,677 participants at baseline in our study which correlates with a similarly reported prevalence of 40.5% is almost the same age group in a recently published study by Hazzaa et al. (22). There has been an increasing trend of adolescent obesity over the past three decades, which has been linked to a parallel increase in chronic health issues such as type 2 diabetes, cardiovascular disease, and MetS in Saudi adolescents (23, 24). Physical inactivity, sedentary behaviors, and unhealthy dietary habits, particularly the popularization and overtake of westernized foods, have been associated with the rising prevalence of obesity and its health effects in adolescents in this part of the world (25, 26). This is alarming and preventive steps through interventions based on lifestyle change counseling in adolescents, particularly in Saudi Arabia, have been limited in the past and need to be scaled up (27, 28). One way of mitigating the problem is to focus on delivering intensive lifestyle change programs in adolescents at high risk of MetS; however, the better way, especially when unhealthy lifestyle behavior is so prevalent and health awareness is low in this age group, is to deliver general health awareness and lifestyle change programs. Lifestyle change programs in high-risk adults have been implemented earlier by our group (29–33). In adolescents, school settings for these lifestyle interventions may prove to be the best setting. In collaboration with SCAD, our group completed this 12-month lifestyle change awareness program based on the educational counseling sessions delivered in the school setting, and the results indicated that it was effective in reducing cardiovascular risk factors in adolescents despite interruptions caused by the COVID-19 pandemic during the program.

School-based settings like one used in the present program are important for behavioral interventions aimed at mitigating the risks of obesity and overweight, as well as increasing nutrition and physical activity levels in children and adolescents because they give an accessible way to reach this age group (34). In some countries such as the United Kingdom, health education delivered during school or after school hours has been part of the government’s strategy to address the rising prevalence of obesity and its health effects in children and adolescents (35). Initiatives like these should be adopted by policymakers and other involved entities, and the health education counseling practices may be included in the normal educational curriculum. Some earlier reviews of lifestyle change interventions based on the school setting showed mixed results of its effectiveness (36, 37); however, recent bigger systematic review and meta-analysis by Liu et al. comprising 50 interventions (38) and by Jacob et al. (39) comprising 33 school-based health education interventions in children and adolescents suggested its effectiveness to lower BMI toward a healthier range. The results of the 12-month counseling-based education program that we did in 60 schools in Riyadh city are consistent with the findings of this meta-analysis since there was a modest but significant overall drop (−4.5%) in the prevalence of central obesity post-intervention.

MetS, a group of cardiometabolic risk factors, is on the rise among adolescents, particularly in developing countries such as Saudi Arabia, and has long-term health effects, including the development of more serious health risks such as type 2 diabetes, endocrine disorders, and cardiovascular disease (10, 40, 41). It is not recommended to apply the adult definition of MetS in children and adolescents in whom the development of the definition has faced the challenges (42); however, researchers agree that more focus should be on recognizing the clustering of cardiometabolic risk factors in children and adolescents rather than on defining whether they have reached the critical cutoff of the diagnosis of MetS (43). Therefore, screening each cardiometabolic risk factor and seeking an improvement in all of them should be the main goal of lifestyle intervention programs for MetS, especially in children and adolescents. Though there are a lot of observational and interventional studies targeted at lowering the risk of obesity in children and adolescents, studies on the lifestyle change program’s effectiveness in reducing metabolic diseases in this age range are limited. However, the few performed before suggest long-lasting benefits of such programs in reducing MetS in children and adolescents (44, 45). Furthermore, because sedentary lifestyles and the risk of cardiometabolic diseases go hand in hand, school-based counseling and physical activity promotion offer an effective intervention in mitigating the risks of MetS in children and adolescents regardless of BMI status, as seen in previous interventions (46, 47). At the same time, we need to look at differences in sociodemographic conditions of the communities to devise such intervention programs targeted at changing the dietary patterns and physical activity levels of the adolescents at risk (48). The results of this counseling-based intervention program in Saudi schools are in line with the results of these interventions and suggest its effectiveness in individual components of MetS, particularly low HDL-cholesterol.

This study found a moderate but insignificant drop in the prevalence of the full MetS phenotype post-intervention, despite certain components decreasing, but a corresponding increase in the prevalence of hypertriglyceridemia in the participants post-intervention. One of the reasons might be the interruption of the planned face-to-face educational counseling delivered at schools post the emergence of COVID-19 restrictions and the closure of schools in March 2020. All the counseling sessions (five per participant) were initially planned to be delivered face-to-face in schools, and the orientation sessions were done as planned; however, most of the follow-up sessions were conducted through virtual meeting platforms, which might have impacted the results. While less is known of the impact of interruptions caused by COVID-19 pandemic on school-based lifestyle interventions, some reports suggest an overall negative impact on such interventions (49, 50). Furthermore, the constraints imposed by COVID-19 on such school-based initiatives may lead to an increase in risk factors such as obesity in children and adolescents owing to an increase in sedentary behaviors and screen time (51, 52). Considering the overall impact of these restrictions, as well as the fact that school closures have increased the tendency of children and adolescents toward an obesogenic environment, an initiative like the current study, which has at least helped in mitigating the rising prevalence of MetS in the study participants over 12 months, may still be considered effective and may be adopted as a model to curb this menace by the relevant authorities.

This study should be interpreted taking into consideration the following limitations. First, the lack of control group limits the findings to at best, suggestive, since the changes observed overtime cannot be directly attributed to the intervention given. Second, we provided education to the study participants in the form of general health awareness counseling in place of intensive monitoring to specific risk groups such as for overweight and obese adolescents. Third, we did not actively monitor dietary and physical activity changes since the intervention was more of guidance rather than intensive approach. The high attrition rate may have limited some aspects of the results, but was justified due to the restrictions imposed during the study program. In future interventions, behavioral counseling such as self-efficacy, goal setting, and feedback should be applied more aggressively.

## Conclusion

Despite COVID-19-related interruption and the overall increase in the prevalence of hypertriglyceridemia and hyperglycemia, the multi-platform, counseling-based health awareness education campaign modestly improved the cardiometabolic status of Arab adolescents, with at least one of the MetS risk components showing a favorable change post-intervention in 2 out of every 3 Arab adolescents. Given the quasi-experimental design, the results should be replicated with the inclusion of a control group. Regardless, interventions like these may have clinical implications in policymaking toward an academic curriculum equipped with awareness in healthy eating and physical education.

## Data Availability Statement

The raw data supporting the conclusions of this article will be made available by the corresponding author as per the guidelines of the institution.

## Ethics Statement

The studies involving human participants were reviewed and approved by Institutional Review Board (IRB) of the College of Medicine, KSU, Saudi Arabia (No. E-19-4239, 29 October 2019). Written informed consent to participate in this study was provided by the participants’ legal guardian/next of kin.

## Author Contributions

NA-D, KW, and SS designed the study. AA and OA worked in the methodology. KW and MK did the statistical analysis. NA, HA, MA, and AH helped in the data curation. KW wrote the manuscript. SS revised the manuscript. NA-D did the study supervision. All authors contributed and approved the final version of the manuscript.

## Conflict of Interest

The authors declare that the research was conducted in the absence of any commercial or financial relationships that could be construed as a potential conflict of interest.

## Publisher’s Note

All claims expressed in this article are solely those of the authors and do not necessarily represent those of their affiliated organizations, or those of the publisher, the editors and the reviewers. Any product that may be evaluated in this article, or claim that may be made by its manufacturer, is not guaranteed or endorsed by the publisher.

## Supplementary Material

Supplementary Material 1 Example of the study materials distributed to the study participants.
