# Supplementary material for: Cardiometabolic Effects of a 12-Month, COVID-19 Lockdown-Interrupted Lifestyle Education Program for Arab Adolescents
Source: Front Pediatr. 2022 Jun 9;10:887138. doi: 10.3389/fped.2022.887138 (PMC9245569; doi:10.3389/fped.2022.887138)
Supplement: Supplementary Material 1 — Example of the study materials distributed to the study participants. [file Data_Sheet_1.PDF]

SCAD
جمعية السكري السعودية الخيرية
Saudi Charitable Association of Diabetes

Diabetes Risk Factors

CBCD
كرسي المؤشرات الحيوية للأمراض المزمنة
CHAIR FOR BIOMARKERS OF CHRONIC DISEASES

Main diabetes risk factors

1. High blood cholesterol level
2. Obesity
3. High blood pressure
4. Sedentary life style

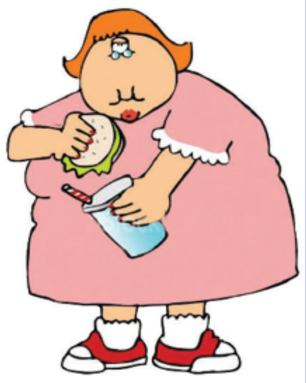

First factor; High blood cholesterol level

Body fats are subdivided into two types;

1. Triglycerides
2. Cholesterol

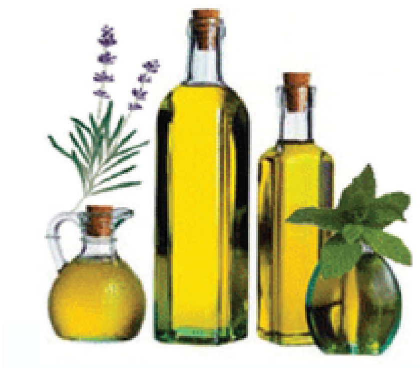

- **Types of cholesterol:**

1. Good cholesterol; HDL which helps the body getting rid of fats. HDL level in men is higher than its level in women, its level increase by exercise and weight loss .
2. Bad cholesterol; LDL which transport fats to body cells and it is the main suspect of high cholesterol complications specially arteries disease and atherosclerosis.

Cholesterol level should be screened in patients more than 20 years old. The patient should be fasting for 10-12 hours. In case of normal range result repeat every 5 years.

- **Sources of triglycerides;**

1. External source: obtained from food

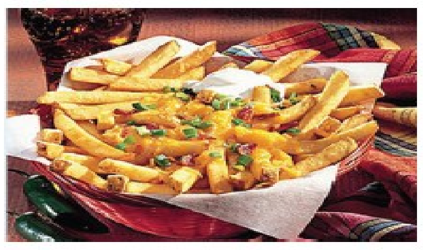

- **Sources of cholesterol;**

1. Internal source: it is synthesized by liver which provides body by 800 mg/day.
2. External source: cholesterol rich meals like animal fats, milk products, egg yolks, red meats, fried food, and fast food.

Avoid eating cholesterol rich meals (more than 200 mg/day).

Egg yolks	150-300 gm cholesterol in one egg
Cattle parts like marrow, brain, spleen, and liver	200-2000 mg cholesterol per 100 gm
Lamb meat	60 gm allowed daily
Butter or cream	250 mg cholesterol per 100 gm

Second factor; Obesity

Increase in body weight over normal range due to fat accumulation being over the ideal body weight by 30%.
This fat accumulation in the body increases the volume and number of fat cells.

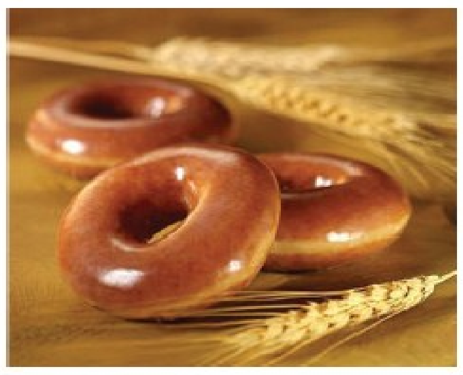

- **Conquering diabetes risk factors:**

1. We can not do any thing about genetic factors and age but we can control body weight and physical activity.
2. Persons above 40 years old and over weight need to measure their blood sugar and if normal repeated every 3 years.

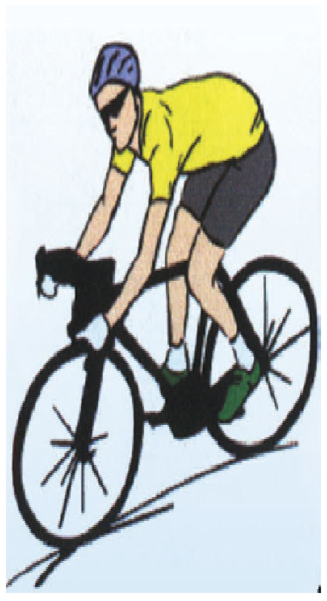

- **Obesity measure:**

Obesity is measured by many ways like;

1. Height weights charts for men and women
2. Calculation of body mass index BMI
3. Measuring waist circumference
4. Waist /hip circumference ratio (apple or bear body shape)

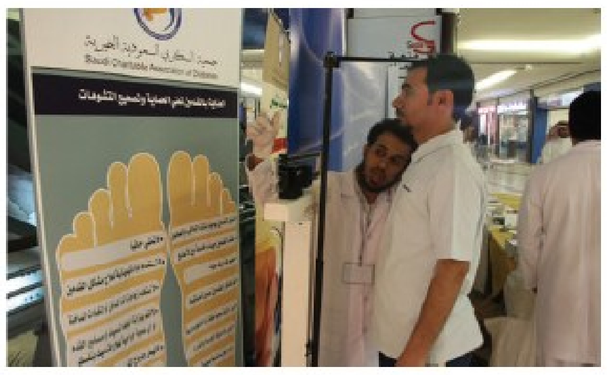

Body mass index	Explanation
Less than 18.5	Less than normal body weight
18.5-24.9	Normal body weight
25-29.9	Overweight
≥ 30	Obese

- **Waist circumference**

It is measured by meter put tightly on waist. It is a good indicator to obesity risk factors.
Risk increase with waist circumference above 90 for women and 100 for men.
Doctors and health team are concerned not only with amount and increase of body fat but also where it accumulates.

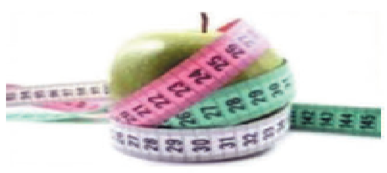

SCAD

جمعية السكري السعودية الخيرية

Saudi Charitable Association of Diabetes

Diabetes Risk Factors

CBCD

كرسي المؤشرات الحيوية للأمراض المزمنة

CHAIR FOR BIOMARKERS OF CHRONIC DISEASES

Fat accumulation in abdomen and circumference called Apple Obesity found more often in men is more dangerous than fat accumulation in hip area called Pear Obesity found more often in women.

- **Types of obesity:**

- First type; Childhood onset obesity: it continues with aging, it is hard to treat because it is due to increase in number of fat cells which can not be decreased by limiting caloric intake.

- Second type; Middle- age onset: it occurs in women more than men, it is due to increase of fat cells volume not number. It can be treated with changing life style and healthy diet.

- **Obesity complications:**

Obesity is not just a cosmetic or psychological problem but also a true health problem. Person with 20% over their ideal body weights have higher risk for many health problems, early-age, and complications death twice persons with normal body weights.

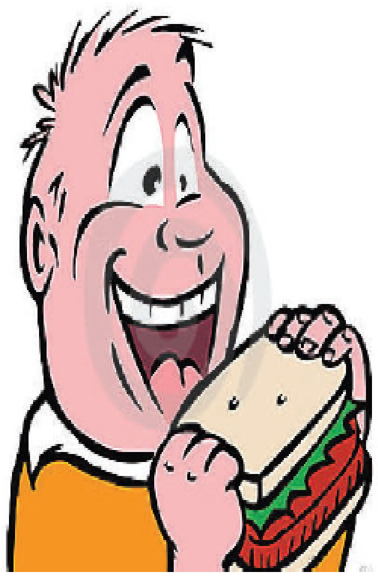

Fat cells affects body in two ways;

First; Increasing need for insulin.

Second; Insulin resistance in obese person result in increase of insulin level in the blood (hyperinsulinaemia), which takes place due to low number of insulin receptors on fat cells and defect in insulin use inside the cell itself.

Weight reduction in patients with type II diabetes decreases insulin resistance and improves blood glucose level control.

-Balance between energy intake and expenditure which is fulfilled by healthy diet and exercise program that suits patient sex, age, weight, type of activity.

In some condition , bariatric surgery is agood option for obesity especially with morbid obesity , or failure of the above steps , you can ask your doctor about it .

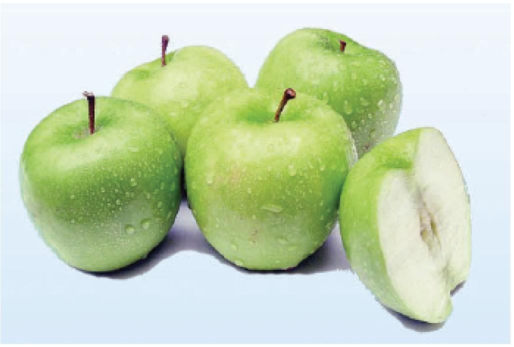

-Exercising continually at least 3 times \week after medical consultation.

Imbalance between energy intake and expenditure is due to:

1. Genetic factor
2. Environmental factor
3. Psychological factors
4. Endocrine diseases

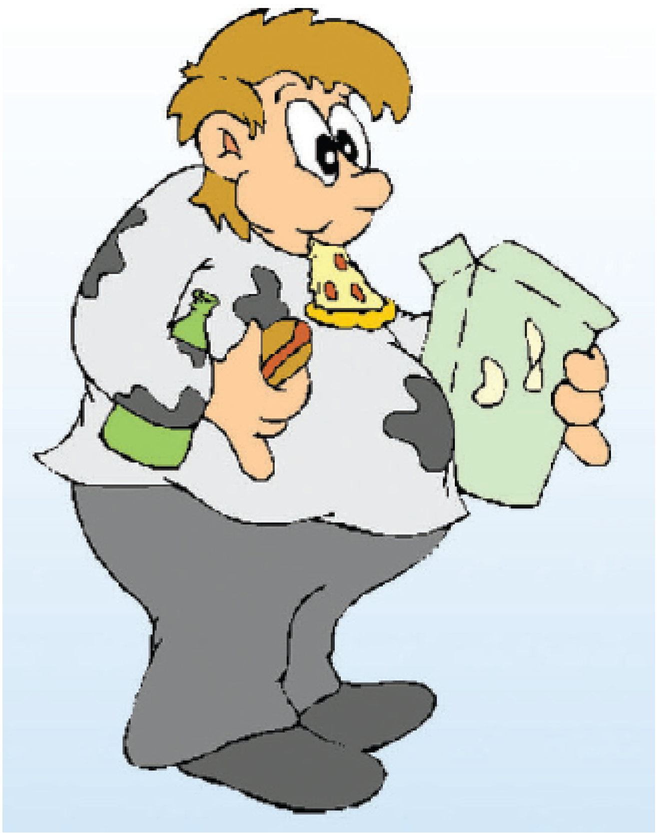

These are some of the complication :-

- Heart and blood vessels diseases (coronary artery diseases)
- High blood cholesterol and triglycerides
- Some cancers (breast, and colon)
- Type II diabetes
- Sleep apnea
- Psychological complications
- High blood pressure
- Gallbladder stones
- Joints pain
- Gout
- Infertility

- **How to control obesity:**

-The most important step that decreasing weight should be self raised not to satisfy others which ends usually with failure.

-Set a reasonable aim which is not always the ideal body weight but it can be reaching normal levels of cholesterol and triglycerides.

-Start life style modification at suitable time because it requires a huge mental effort to keep up and succeed.

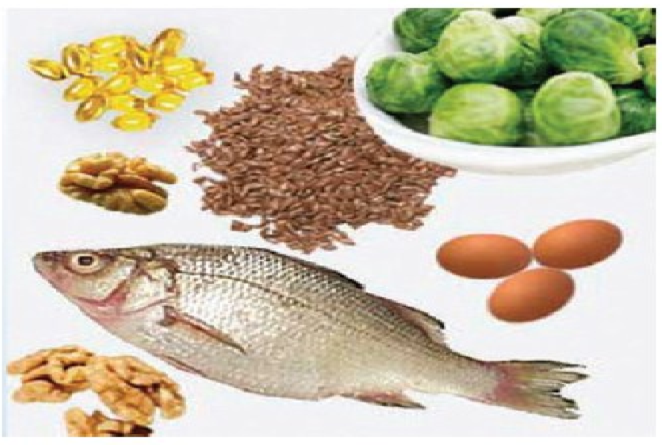

-Loosing weight is a slow process that needs patience and endurance to keep up and

-Know your bad eating habits high fat intake, eating in front of television, and not eating enough fruits and vegetables.

-Make a notebook for a week meals to write down types and amount of food to find out what is your weak points and bad obesity-causing habits.

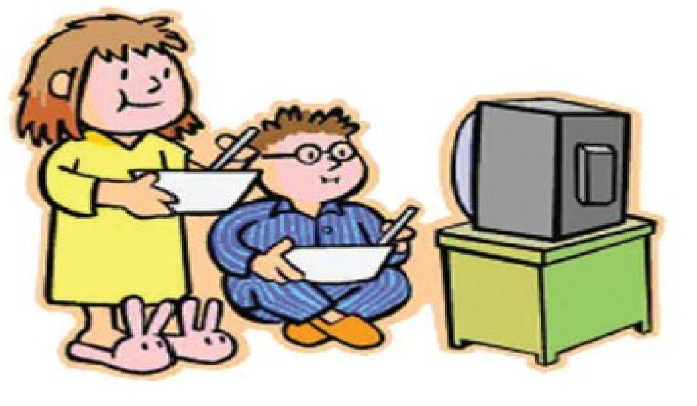

-Deep and continual behavioral change in eating and getting used to increased physical activity.

جمعية السكري السعودية الخيرية
Saudi Charitable Association of Diabetes

SCAD

Diabetes Risk Factors

CBCD

كرسي المؤشرات الحيوية للأمراض المزمنة
CHAIR FOR BIOMARKERS OF CHRONIC DISEASES

- **Third factor; High blood pressure**

Blood pressure is the forcing of blood by heart into arteries carrying oxygen and nutrients to different body tissues and organs and blood back to heart nonoxygenated via veins and so heart pump it to lungs to reoxygenate it and send it back to heart to pump it to arteries again.
High blood pressure is the increase of pressure on the arteries wall leading to atherosclerosis (loosing elasticity of the walls) which makes it hard to pump the blood.

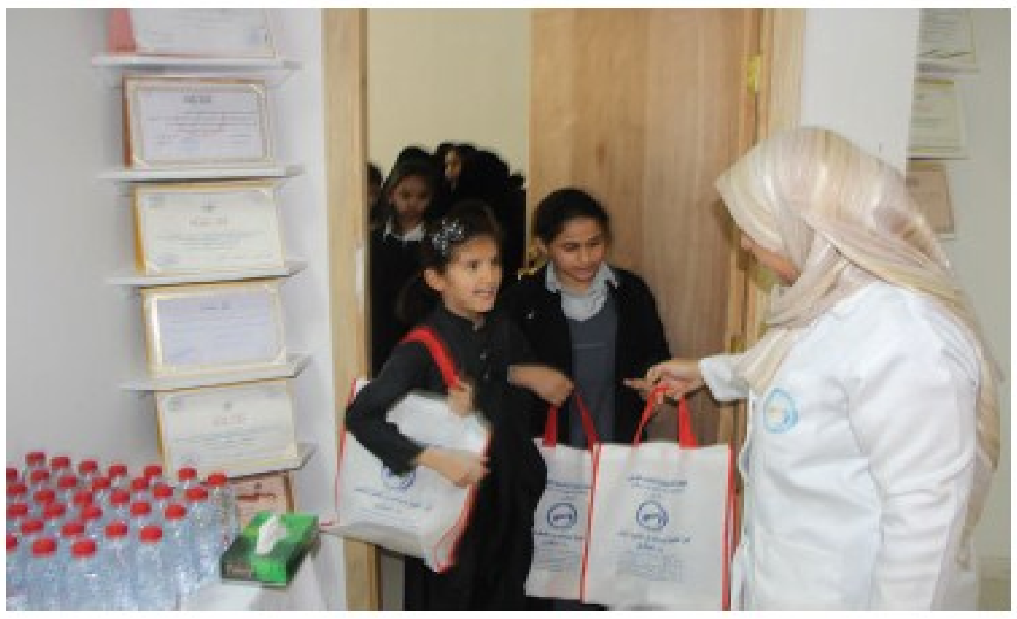

- **High blood pressure treatment:**

Non drug approach:
1. Overcome stress
2. Physical activity
3. Potassium rich foods
4. Tea and coffee reduction
5. Lower salt intake.
6. Stop smoking
7. Body weight reduction

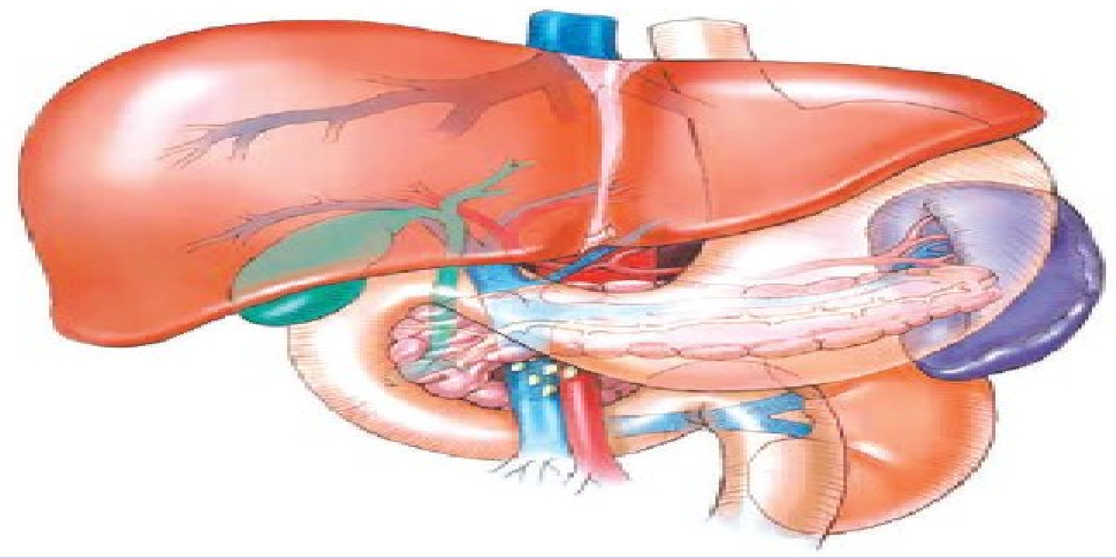

What is normal blood sugar level?

70-100 mg/dl before eating

Eating carbohydrates like potato, sugar, bread, and biscuits because they are digested and absorbed fast to the blood as glucose when insulin should work to transform it to energy

When pancreas fail to secrete insulin blood sugar level increases which makes patient feels symptoms such as frequent urination, general fatigue, and thirst.

Diabetes affect organs

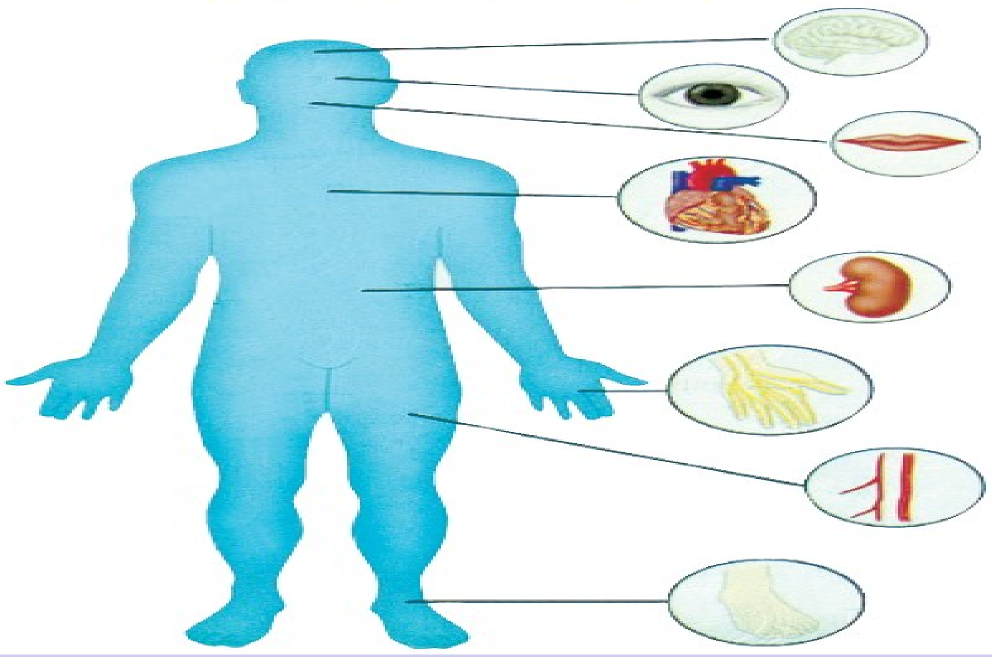

Types of diabetes

A. Type I diabetes;
It is when pancreas is not able to produce insulin which happens usually in childhood. It was called insulin dependent diabetes mellitus (IDDM) and it depends on insulin in treatment.

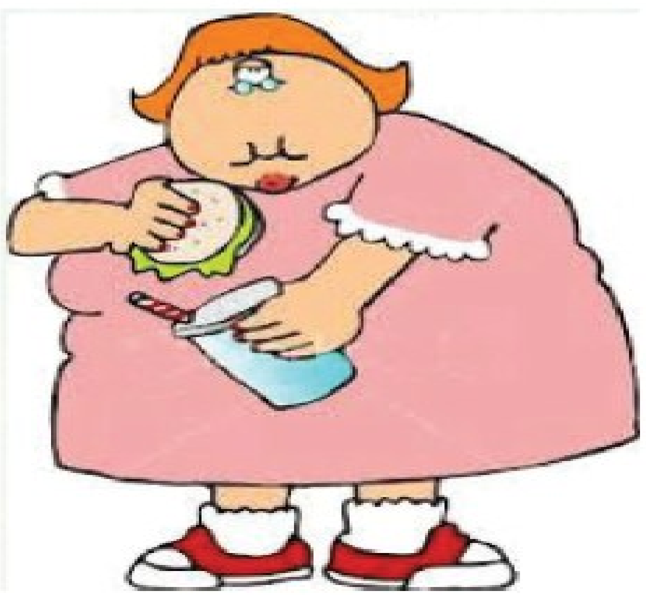

B. Type II diabetes;
It is when pancreas is not secreting enough insulin. It happens usually in adults especially obese or over weight.

Weight reduction or using some drugs that increases activity of pancreas or may need insulin treatment. It is called insulin independent diabetes mellitus.

Symptoms of hyperglycemia: (high glucose level)

1. Frequent urination
2. Thirst
3. Dry skin and mouth
4. Hunger
5. Weight loss
6. Tiredness, fatigue
7. Frequent infections
8. Blurred vision

Symptoms of hypoglycemia: (low glucose level)

1. Sweating and shivering.
2. Fast heart beat.
3. Sleepiness
4. Jerking movement
5. Confusion and poor concentration and talking in a strange way
6. Change in attitude like aggressiveness, and crying
7. Coma

جمعية السكري السعودية الخيرية
Saudi Charitable Association of Diabetes

healthy diet

كرسي المؤشرات الحيوية للأمراض المزمنة
CHAIR FOR BIOMARKERS OF CHRONIC DISEASES

Healthy nutrition

Nutrition definition:

Food is what the body need daily in different life stages to provide the body with energy and keep its tissues healthy. Healthy diet should contain the following elements; Proteins, carbohydrates, fats, vitamins, minerals and water.

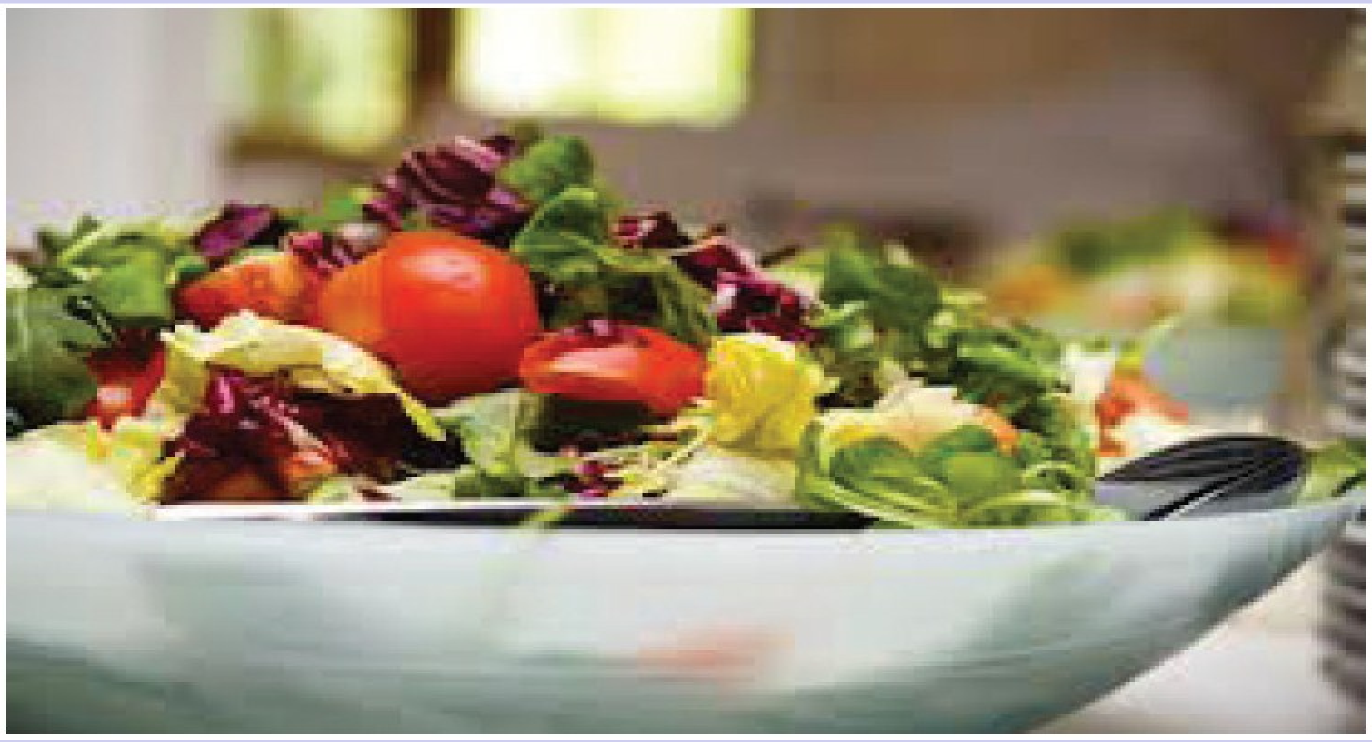

Nutrients` sources

First; Protein:

It is the most important element in construction and maintenance of tissues and muscles and the most effective part in energy production and these are;

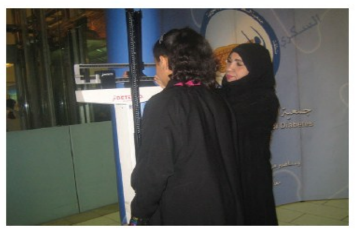

A. Animal protein sources; like eggs, meat, fish and poultry, milk and milk products
B. Plant protein sources; legumes like lentils, beans and chick peas

Third; fats and its sources:

It is important for life and health but if increased above the necessary amounts it will be dangerous.

Sources;

A. Animal source like butter, ghee, and lard
B. Plant source like oils expressed form sees like sesame, olive and corn oil.

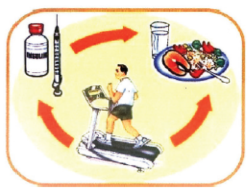

Diet balance for good health

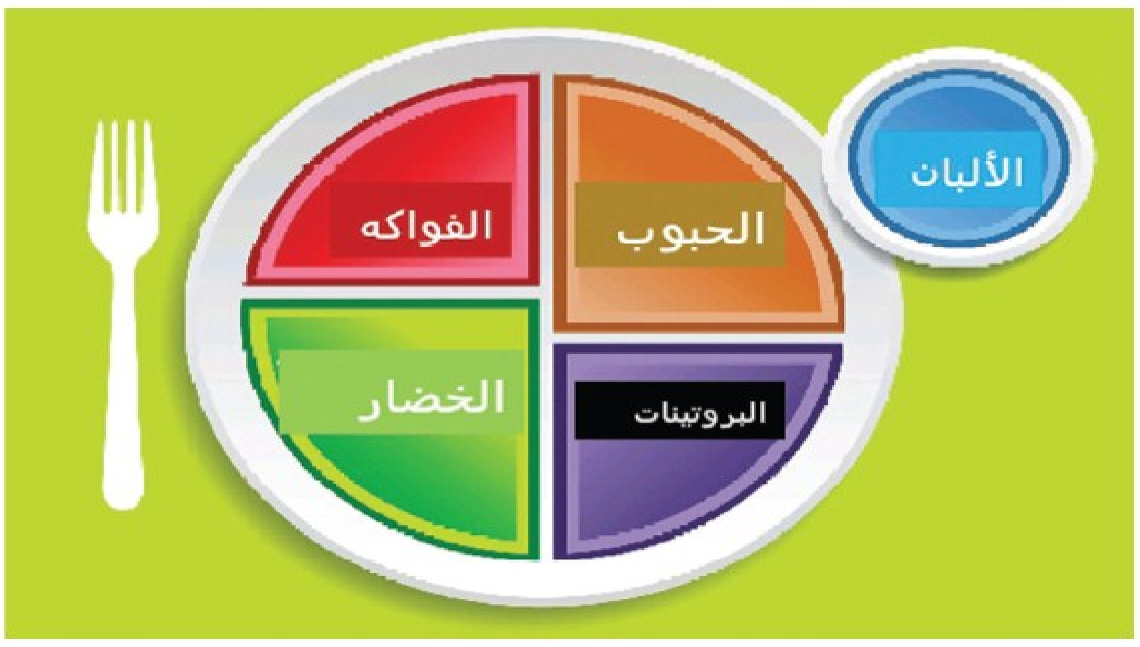

Second; Carbohydrates:

Sources of carbohydrates and sugars;

A. Complex carbohydrates; found in vegetables, bread, potato, rice, pastries , complex carbohydrates is essential .
B. Simple carbohydrates; sugar, honey, jam, juices

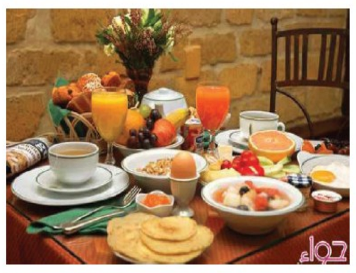

Fourth; Minerals:

Contribute to body formation in muscles and tissues, glands, red and white blood cells.

Fifth; Vitamins:

Important for body maintenance, growth and disease prevention, and deficiencies can cause a lot of diseases.

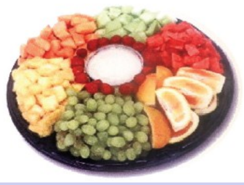

Seventh; Condiments and spices:

Condiments and spices give food a good flavor and taste which acts as an appetizer and a good food aroma like (cardamom, cumin, and cinnamon. They affect stomach directly and activate it by increasing its movement, they also affect taste nerves and hence the increase of gastric juices. Over using spices and condiments is considered bad for health especially hot spices because it causes stomach ulcers and affects kidney and liver as well.

Sixth; Water:

Water is an important body component that contributes to formation of cells and tissues and blood cells, it also dissolves waste products and excrete it in urine and sweat. It regulates body temperature.

Importance of food diversity

It grantees food balance by eating all food elements necessary for the body by eating daily suitable quantities of each element.

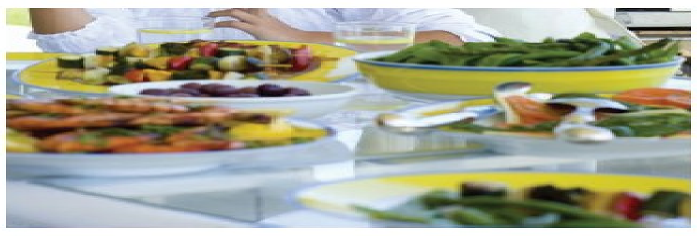

There is no such a complete food with all elements but all kinds of food are complementary to each other.

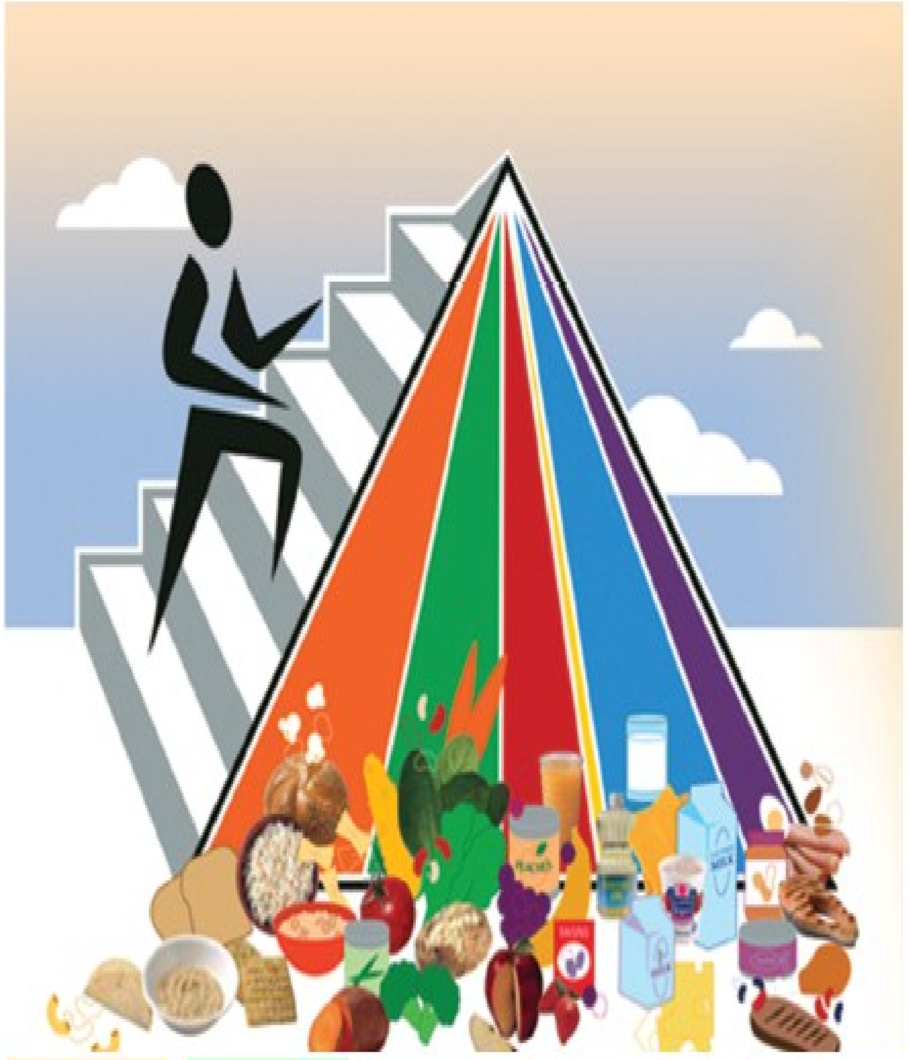

Complex carbohydrates	Vegetables	Fruits	Milk and milk products	Meats and beans
•Eat whole wheat grains like whole grain bread, wheat, grit, corn, and barley.	•Eat dark green vegetables like spinach, molokhia, radish, arugula, celery, parsley, and others<br>•Eat orange colored vegetables like carrot and pepper.	•Eat diversity of fruits.<br>•Eat whole fruits instead of only its juices.<br>•Avoid artificial sweetened juices.	•Choose low fat or skimmed milk or milk products	•Eat meat, poultry, and fish after removing their skin.<br>•Recommended<br>•Cooking methods are boiling, grilling, and oven cooking instead of frying.<br>•Eat diversity of proteins not only few kinds.<br>•Eat legumes like beans, lentil, black-eyed beans, and kidney beans as an alternative of meat group

Healthy ways to preserve food:

1. Keep milk in the fridge away from light.
2. Toast your bread at low temperature to avoid loosing vitamins.
3. Avoid either over washing rice and cooking it with too much water.
4. Eat fresh fruits and vegetables after good wash.
5. Vegetables with leaves like spinach and lettuce are wrapped with wet tissue after washing and kept in cool place. While tomatoes, peas, and green beans are kept in lower temperature
6. Green tomatoes are kept at room temperature and light to ripen.
7. Orange and citrus fruits are kept at room temperature while its juices are kept in fridge in sealed vessels.
8. Cook potato unpeeled after good wash and peel it after cooking.

Out door meal

Fried chicken meal

3 chicken pieces
Medium French fries
Medium coke

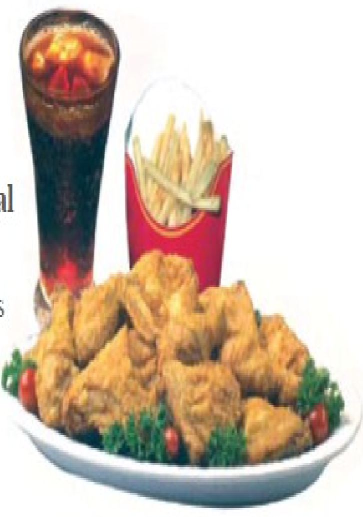

Contains 1249 calories

Exercise needed to burn this meal;
12 hours continuous walking
4 hours continuous swimming

Home made meal

A meal composed of:

12 tablespoons rice
Green salad dish
60 gm grilled meat
A cup of low fat yoghurt
An orange

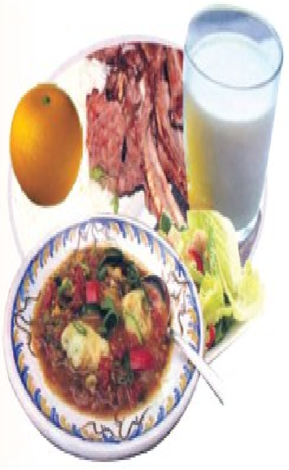

This meal contains 500 calories

It needs a physical activity to consume these calories
5 hours minutes continuous walking
One and half hours continuous swimming

جمعية السكري السعودية الخيرية
Saudi Charitable Association of Diabetes

Exercise

كرسي المؤشرات الحيوية للأمراض المزمنة
CHAIR FOR BIOMARKERS OF CHRONIC DISEASES

Introduction

Exercise is an important part of healthy and ill people lives for keeping the first one as healthy and the later to achieve cure.

Following an exercise schedule will affect greatly in delaying diseases of heart, arteries, blood vessels, and joints deformity so exercise is an important approach to disease prevention or it is the most important.

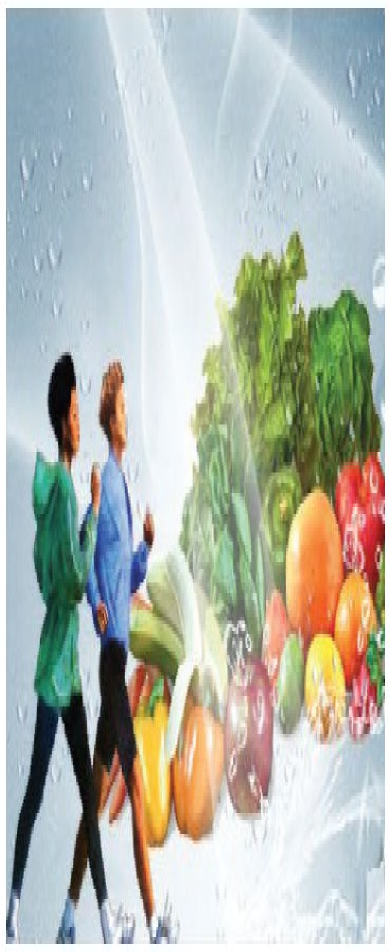

Begin exercising come on .....Come on.

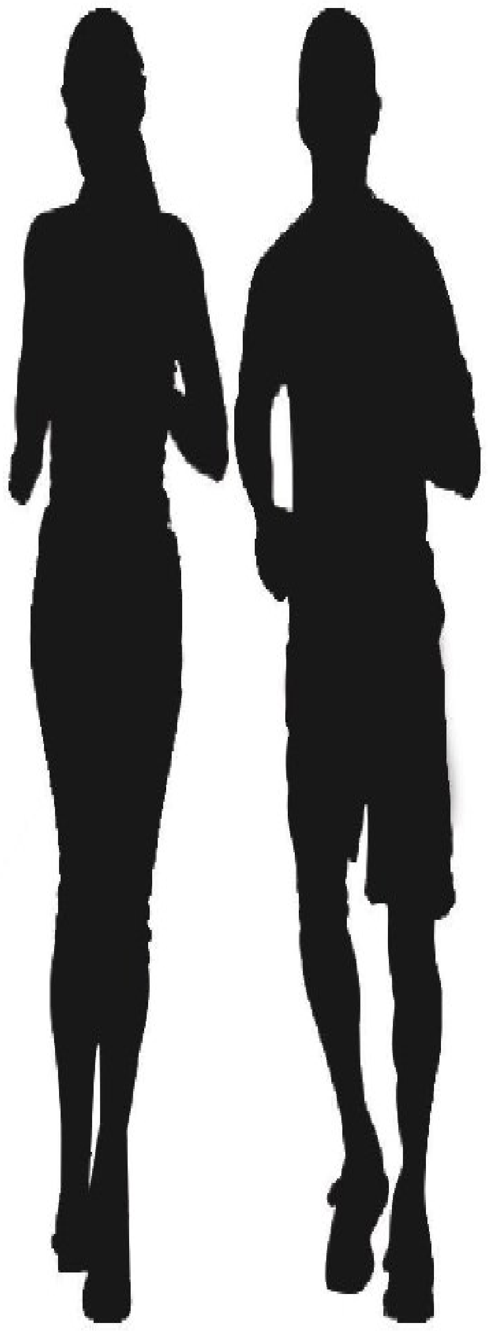

Exercise benefits:

It achieves the following health benefits;

- Better healthy life
- Fitness
- Balanced body shape
- Self respect

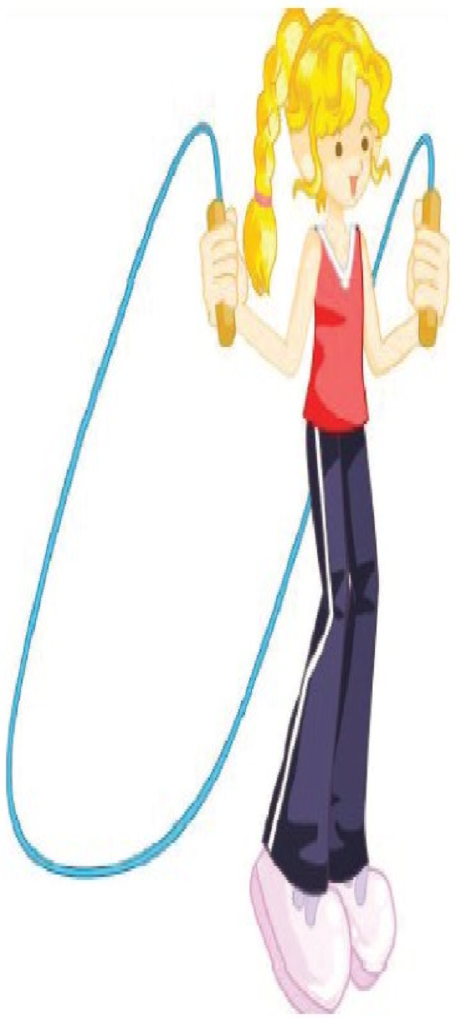

When exercising you should know that:

You should exercise, on regular basis, for at least 5 times per week or daily for 20-30 minutes at least drinking enough amount of water for hydration and avoiding dehydration.

- Control of body weight
- Stronger bones and muscles
- Feeling active and vital
- Less stress

Make exercise a part of your daily life schedule

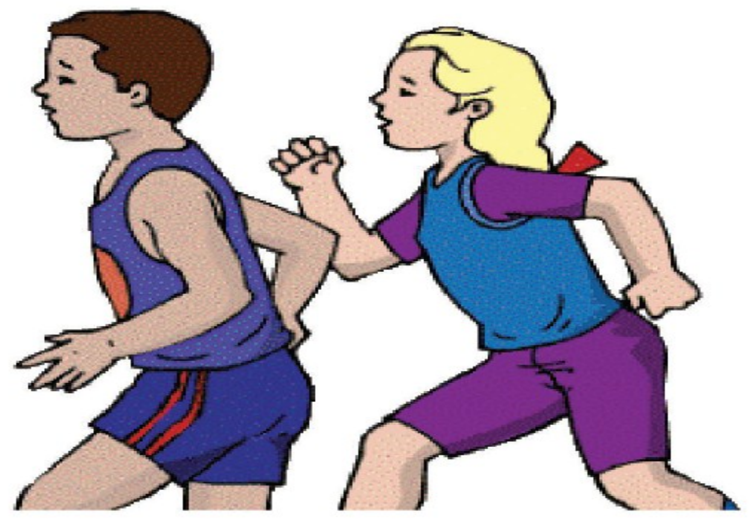

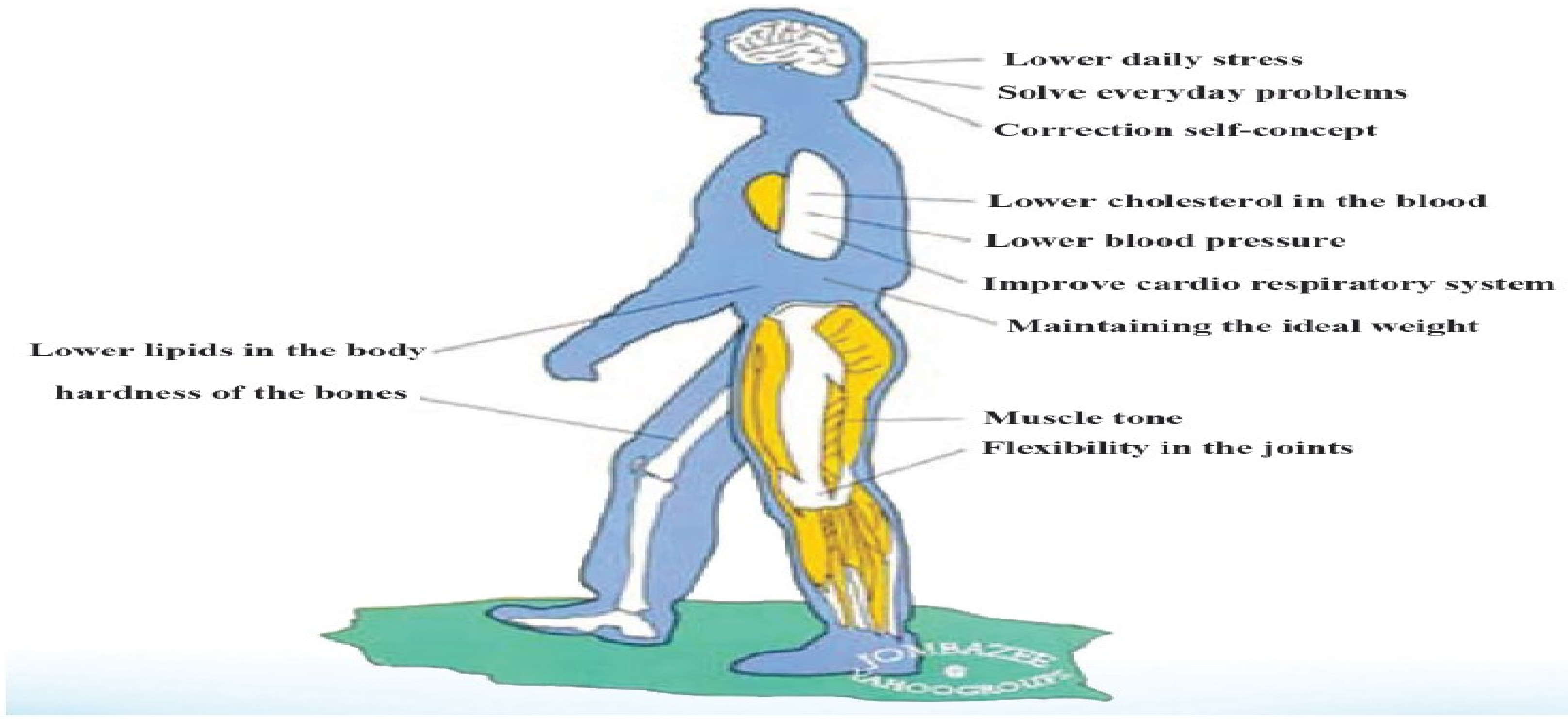

Advice and guidance concerning exercise:

1. You should consult your doctor to perform necessary tests.
2. Following the medicine advice for your doctor
3. Persisting on keeping exercising
4. Choice of suitable time for exercise preferably early morning or sunset
5. Choice of open air places like clubs and gardens for exercise
6. Avoid burning sun or cold weather also fumes and smoke
7. Avoid eating rich meals before exercise with less than two hours
8. Drinking water

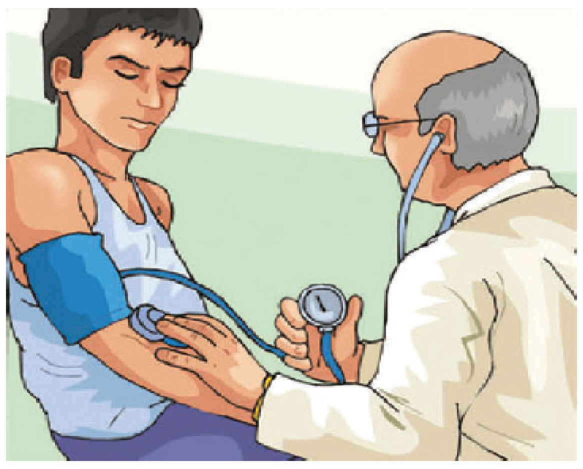

Risks of lack of physical activity:

- Indolence
- Fatigue with the smallest effort
- Anxiety, stress, and insomnia
- Engaging in bad habits such as smoking
- Diseases in early age such as diabetes, high blood pressure, heart and arteries diseases, and obesity and its complications
- Osteoporosis
- Constipation and less intestinal movement
- Early aging ....And others.....
